# Supplementary material for: Immunogenicity and safety of co-purified diphtheria, tetanus and acellular pertussis vaccine in 6-year-old Chinese children
Source: Nat Commun. 2025 Nov 29;17:214. doi: 10.1038/s41467-025-66890-7 (PMC12780235; doi:10.1038/s41467-025-66890-7)
Supplement: Supplementary file 1 — Supplementary information [file 41467_2025_66890_MOESM1_ESM.pdf]

# Supplementary information


| Contents | Page |
|---|---|
| **Supplementary Table 1.** Seroconversion rates to co-purified DTaP or DT before and day 28 after vaccination based on subgroups that were positive or negative for antibodies at baseline. | 2 |
| **Protocol** | 3-18 |
| **CONSORT 2010 checklist** | 19-20 |

**Supplementary Table 1.** Seroconversion rates to co-purified DTaP or DT before and day 28 after vaccination based on subgroups that were positive or negative for antibodies at baseline.

| **Baseline antibody** | **co-purified DTaP** | | **DT** | | ***P*** |
|---|---|---|---|---|---|
| | **N** | **Rate, n (%)** | **N** | **Rate, n (%)** | |
| **anti-PT** | | | | | |
| ⩾20 IU/ml | 21 | 11(52.38) | 8 | 0(0) | |
| < 20 IU/ml | 213 | 179 (84.04) | 216 | 5 (2.31) | |
| **anti-FHA** | | | | | |
| ⩾20 IU/ml | 85 | 31 (36.47) | 69 | 0(0) | |
| < 20 IU/ml | 149 | 143 (95.97) | 155 | 8 (5.16) | |
| **anti-DT** | | | | | |
| ⩾0.1 IU/ml | 87 | 87 (100.00) | 77 | 77 (100.00) | 1.000 |
| < 0.1 IU/ml | 147 | 147 (100.00) | 147 | 147 (100.00) | 1.000 |
| **anti-TT** | | | | | |
| ⩾0.1 IU/ml | 214 | 207 (96.73) | 203 | 174 (85.71) | <0.001 |
| < 0.1 IU/ml | 20 | 20 (100.00) | 21 | 21 (100.00) | 1.000 |

The two-sided $\chi2$ test or Fisher's exact test to compare seroconversion rates between arms. anti-PT, anti-pertussis toxin antibody; anti-FHA, anti-filamentous hemagglutinin antibody; anti-DT, anti-diphtheria antibody; anti-TT, anti-tetanus antibody; co-purified DTaP, co-purified diphtheria, tetanus and acellular pertussis combined vaccine; DT, diphtheria and tetanus combined vaccine.

# A randomized, controlled, clinical trial of the booster dose of co-purified diphtheria, tetanus and acellular pertussis combined vaccine (co-purified DTaP) or diphtheria and tetanus combined vaccine (DT) at Children Aged 6 Years

Registration Class: Prophylactic biological product

Clinical Phase: IV

Protocol No.: DTaP/DT-2023-02

Version No.: 1.0

Version Date: April 17, 2023

Sponsors (seal): China National Biotec Group Company Limited

Chengdu Institute of Biological Products Co., Ltd.

Wuhan Institute of Biological Products Co., Ltd.

Research Institutions: Zhejiang Provincial Center for Disease Control and Prevention

## RESEARCH TEAM

| **Protocol No.** | DTaP/DT-2023-02 | **Version No.** | 1.0 |
|---|---|---|---|
| **Study Title** | A randomized, controlled, clinical trial of the booster dose of co-purified diphtheria, tetanus and acellular pertussis combined vaccine (co-purified DTaP) or diphtheria and tetanus combined vaccine (DT) at Children Aged 6 Years | | |
| **Leading Clinical Research Institution** | | | |
| Name: Zhejiang Provincial Center for Disease Control and Prevention<br>Address: 3399 Binsheng Road, Binjiang District, Hangzhou<br>Tel: 0571-87115170<br>Postal code: 310051<br>Department: Immunization Program<br>Department director: Hanqing He | | | |
| **Principal Investigator** | | | |
| Institution: Zhejiang Provincial Center for Disease Control and Prevention<br>Name: Hanqing He<br>Tel./Mobile: 0571-87115170<br>E-mail: hanqinghe@cdc.zj.cn | | | |
| **On-Site Clinical Research Institutions** | | | |
| Site 1<br>Institution name: Fuyang District Center for Disease Control and Prevention<br>Address: No. 429 Beihuan Road, Fuyang District, Hangzhou<br>Postal code: 311400<br>Director: Junjie Jin<br>Tel: (86)15888827937<br>E-mail: 2546803342@qq.com<br>Site 2 | | | |

| |
|---|
| Institution name: Tongxiang City Center for Disease Control and Prevention<br>Address: No. 1238 Wenhua Street, Tongxiang City, Jiaxing<br>Postal code: 314500<br>Director: Jinwei Wu<br>Tel: (86)18858311061<br>E-mail: 759716707@qq.com |
| **Monitors/Auditors** |
| Institution name: China National Biotec Group Company Limited<br>Address: No. 2 Shuangqiao Road, Chaoyang District, Beijing<br>Tel: 13381186408<br>Contact: Linyun Luo<br><br>Institution name: Chengdu Institute of Biological Products Co., Ltd.<br>Address: No. 379, 3rd Section, Jinhua Road, Jinjiang District, Chengdu, Sichuan<br>Tel: (86)15882123758<br>Contact: Wei Zhao<br><br>Institution name: Wuhan Institute of Biological Products Co., Ltd.<br>Address: No. 1 Huangjingongyeyuan Road, Jiangxia District, Wuhan<br>Tel: (86)15207190297<br>Contact: Jiayou Zhang |
| **Technical Collaborators** |
| Institution name: National Institutes of Food and Drug Control<br>Address: No. 31 Huatuo Road, Daxing District, Beijing<br>Contact: Xiao Ma<br>Tel: 010-67095346 |
| **Statistical Analysis** |
| Institution name: Harvest Integrated Research Organization (HiRO) |

Address: No.1800 Panyuangong Road, Chongming District,Shanghai

Contact: Lianying Zhao

Tel: (86)13810891050

# PROTOCOL SYNOPSIS

| | |
|---|---|
| **Protocol Name** | A randomized, controlled, clinical trial of the booster dose of co-purified diphtheria, tetanus and acellular pertussis combined vaccine (co-purified DTaP) or diphtheria and tetanus combined vaccine (DT) at Children Aged 6 Years |
| **Product Features** | (1) Co-purified diphtheria, tetanus and acellular pertussis combined vaccine (co-purified DTaP) is made of acellular pertussis vaccine stock solution, diphtheria toxoid stock solution and tetanus toxoid stock solution plus aluminum hydroxide adjuvant. It is a kind of white or light yellow suspension. After placing, the adjuvant sinks and shakes to form a uniform suspension. Contain appropriate amount of thiomersal bacteriostatic agent.<br>(2) diphtheria and tetanus combined vaccine (DT) made from diphtheria toxoid stock solution and tetanus toxoid stock solution plus aluminum hydroxide adjuvant. It is a milky white uniform suspension. The adjuvant should be placed for a long time to sink. The upper layer of the solution should be colorless and clear, but it can be evenly dispersed after shaking, containing an appropriate amount of thiomersal bacteriostatic agent. |
| **Indications /Study Population** | The co-purified DTaP is applicable to children aged 3 months to 6 years.<br>The DT is applicable to children under 12 years of age. |
| **Study Objective** | The safety and immunogenicity of fifth dose of co-purified DTaP against pertussis in 6-year-old children. |
| **Study Design** | This trial uses a randomized, controlled, open-label design to evaluate the safety and immunogenicity of the booster dose of co-purified DTaP or DT at children aged 6 years.<br>This study plans to enroll 480 subjects who will be randomized into 2 groups with 240 subjects per group.<br>**<u>Grouping:</u>**<br>Study group is the co-purified DTaP group: The target number of |

| | |
|---|---|
| | enrollment is 240. Blood samples will be collected before immunization on D0 and the subjects will then be vaccinated with the co-purified DTaP (0.5 mL/vaccine); blood samples will be collected on D28.<br>Control group is the DT group: The target number of enrollment is 240. Blood samples will be collected before immunization on D0 and the subjects will then be vaccinated with the DT (0.5 mL/vaccine); blood samples will be collected on D28. |
| **Hypotheses** | 1.The seroconversion rate of pertussis antibody in 6-year-old children vaccinated with co-purified DTaP vaccine is not less than 70%.<br>2. There was no significant difference in the seroconversion rates of diphtheria and tetanus antibodies between 6-year-old children vaccinated with co-purified DTaP vaccine and DT. |
| **Vaccines** | **Vaccine 1: co-purified DTaP**<br>Manufacturer: Chengdu Institute of Biological Products Co., Ltd.<br>Strength: 0.5 mL/vial/dose, the titer of acellular pertussis vaccine, diphtheria vaccine and tetanus vaccine shall not be lower than 4.0 IU, 30 IU and 40 IU respectively.<br>Vaccine lot no.: 20220311<br>**Vaccine 2: DT**<br>Manufacturer: Wuhan Institute of Biological Products Co., Ltd.<br>Strength: 0.5 mL/vial/dose, the titer of diphtheria toxoid is not less than 30IU; the titer of tetanus toxoid should not be lower than 40IU.<br>Vaccine lot no.: 202112020-2 |
| **Duration** | The entire clinical trial lasts about 18 months |

## 1. STUDY OBJECTIVE

### 1.1. Primary objective

The primary outcomes of the study were the seroconversion rates and seropositive rates of anti-pertussis antibodies in the co-purified DTaP group, as well as a comparison of seroconversion rates and seropositive rates of anti-diphtheria and anti-tetanus antibodies in the co-purified DTaP and DT groups.

### 1.2 Secondary objective

The secondary outcomes of the study were the seroconversion rates and seropositive rates of anti-pertussis antibodies in the co-purified DTaP group, as well as a comparison of seroconversion rates and seropositive rates of anti-diphtheria and anti-tetanus antibodies in the co-purified DTaP and DT groups.

## 2. RESEARCH INSTITUTIONS

### 2.1 Clinical Research Institution

Zhejiang Provincial Center for Disease Control and Prevention.

### 2.2 On-Site Clinical Research Institutions

Fuyang District Center for Disease Control and Prevention, Hangzhou

Tongxiang City Center for Disease Control and Prevention, Jiaxing

## 3. TRIAL DESIGN

### 3.1 Design

This trial uses a randomized, controlled, open-label design to evaluate the safety and immunogenicity of the booster dose of co-purified DTaP or DT at children aged 6 years.

This study plans to enroll 480 subjects who will be randomized into 2 groups with 240 subjects per group.

**Randomization:**

The participants were randomly assigned (1:1) to one of two group schedules using a random number table by block randomization. There are a total of 60 blocks with a block length of 8. All eligible subjects were assigned a random number which was used to identify all procedures performed after the subjects had been randomly grouped. Once a random number had been assigned to one subject, it could not be reassigned to another subject.

**Grouping:**

Study group is the co-purified DTaP group: The target number of enrollment is 240. Blood samples will be collected before immunization on D0 and the subjects will then be vaccinated with the co-purified DTaP (0.5 mL/vaccine); blood samples will be collected on D28.

Control group is the DT group: The target number of enrollment is 240. Blood samples will be collected before immunization on D0 and the subjects will then be vaccinated with the DT (0.5 mL/vaccine); blood samples will be collected on D28.

**3.2 Blood Collection**

Two blood samples are collected from each subject for serum antibody testing.

**Pre-immunization blood collection:** A total of 3.0 mL of venous blood will be collected from all subjects before immunization. The isolated serum samples will be aliquoted into 2 tubes (with at least 0.6 mL serum/tube) and stored at -20°C or below.

**Post-immunization blood collection:** A total of 3.0 mL of venous blood will be collected on D28 after the second dose. The isolated serum samples will be aliquoted into 2 tubes (with at least 0.6 mL serum/tube) and stored at -20°C or below. The time window for blood collection is +14 d.

**3.3 Immunogenicity Assessment**

Concentrations of antibodies against pertussis, diphtheria, and tetanus were measured by the Enzyme-Linked Immunosorbent Assay (ELISA) method.

**Immunogenicity assessment criteria for pertussis vaccine:**

Positive: Antibody titer is ≥0.1 IU/ml.

Seroconversion: Antibody concentration is <0.1 IU/ml before immunization and ≥0.1 IU/ml after immunization; or antibody concentration is ≥0.1 IU/ml before immunization and is increased ≥4-fold after immunization.

Antibody level: Geometric mean concentrations (GMCs) of serum antibody before and after pertussis vaccine immunization as well geometric mean increase (GMI) in serum antibody after immunization.

**Immunogenicity assessment criteria for diphtheria vaccine or tetanus vaccine:**

Positive: Antibody titer is ≥20IU/ml.

Seroconversion: Antibody concentration is <20 IU/ml before immunization and ≥20 IU/ml after immunization; or antibody concentration is ≥20 IU/ml before immunization and is increased ≥4-fold after immunization.

Antibody level: Geometric mean concentrations (GMCs) of serum antibody before and after pertussis vaccine immunization as well geometric mean increase (GMI) in serum antibody

after immunization.

### 3.4 Safety Data Collection

Subjects are observed for 30 min after immunization. Diary cards are distributed after immunization and the subjects must record all solicited and unsolicited adverse events (AEs) observed from D0 to D28 post-immunization in the diary cards every day. Adverse events following immunization (AEFI) will be collected by the investigator using the AEFI monitoring system at 6 months after full immunization. Serious adverse events (SAEs) will also be passively collected at 6 months after full immunization.

**Solicited AEs:**

All solicited AEs observed within 7 d of immunization are assumed by default to be related to the vaccine. The relationship between all unsolicited AEs/SAEs and the vaccines will be determined by the investigator.

✓ Local reactions

Pain, induration, swelling, rash, redness, and pruritus.

Systemic reactions

Fever, fatigue/weakness, decreased appetite, nausea, vomiting, diarrhea, allergy, myalgia, and arthralgia.

### 3.5 Adverse Event Grading Criteria

Post-immunization systemic and local reactions are assessed in accordance with the Guidelines for Classification of Adverse Events in Clinical Trials of Preventive Vaccines (2019 edition) by National Medical Products Administration.

**Table 1. Local reaction grading scale**

| Symptom/Sign | Grade 1 | Grade 2 | Grade 3 | Grade 4 |
|---|---|---|---|---|
| **Pain** | Does not affect or slightly affects activities of the limbs | Affects activities of the limbs | Affects activities of daily life | Loss of self-care abilities or hospitalization |
| **Induration and swelling** | Diameter of <2.5 cm | Diameter of ≥2.5cm or area of <50 $cm^2$ | area of ≥50 $cm^2$ or ulcers or secondary infections or phlebitis or aseptic abscess or wound drainage | Abscess, exfoliative dermatitis, dermal or deep tissue necrosis |

| | | | | |
|---|---|---|---|---|
| **Rash and redness** | Diameter of <2.5 cm | Diameter of ≥2.5cm or area of <50 cm$^2$ | area of ≥50 cm$^2$ or ulcers or secondary infections or phlebitis or aseptic abscess or wound drainage | Abscess, exfoliative dermatitis, dermal or deep tissue necrosis |
| **Pruritus** | Pruritus at the injection site that resolves within 48 on its own or after treatment | Pruritus at the injection site that does not resolve within 48 h after treatment | Affects activities of daily life | NA |

**Table 4. Systemic reaction grading scale**

| Symptom/Sign | Grade 1 | Grade 2 | Grade 3 | Grade 4 |
|---|---|---|---|---|
| **Fever *** [axillary temperature (°C)] | 37.3 to <38.0 | 38.0 to <38.5 | 38.5 to <39.5 | ≥39.5 for 3 d |
| **fatigue/weakness** | Does not affect activities of daily life | Slightly affects activities of daily life | Severe myalgia, severely affects activities of daily life | Emergency care or hospitalization |
| **decreased appetite** | Decreased appetite, but not reduced food intake | Appetite decreased and food intake decreased, but body weight did not decrease significantly | Decreased appetite and weight | Interventions are needed (such as gastric tube feeding, parenteral nutrition) |
| **Nausea** | Transient (<24 h) or intermittent and normal food intake | Persistent nausea leading to decreased food intake (24–48 h) | Persistent nausea leading to nearly no food intake (>48 h) or requires intravenous rehydration | Life-threatening (such as hypotension shock) |
| **Vomiting** | 1–2 times/24 h and does not affect activity | 3–5 times/24 h or limited activities | >6 times/24 h or requires intravenous rehydration | Requires hospitalization or other routes of nutrient delivery due to hypotension shock |
| **Diarrhea** | Mild or transient, 3–4 times/d, abnormal stool property, or mild diarrhea that persists for less than 1 week | Moderate or persistent, 5–7 times/d, abnormal stool property, or diarrhea that lasts >1 week | >7 times/d, abnormal stool property, or hemorrhagic diarrhea, orthostatic hypotension, electrolyte | Hypotension shock, requires hospitalized treatment |

| | | | | |
|---|---|---|---|---|
| | | | imbalance, requires >2 L intravenous infusion | |
| **Acute allergic reactions** | Local urticaria (blister), does not require treatment | Local urticaria, requires treatment or mild angioedema, does not require treatment | Extensive urticaria or angioedema requiring treatment or mild bronchospasm | Anaphylactic shock or life-threatening bronchospasm or laryngeal edema |
| **Myalgia (non-injection site)** | Does not affect activities of daily life | Slightly affects activities of daily life | Severe myalgia, severely affects activities of daily life | Emergency care or hospitalization |
| **Arthralgia** | Mild pain, does not affect function | Moderate pain; requires analgesics and/or pain affects function but not activities of daily life | Severe pain; requires analgesics and/or pain affects activities of daily life | Disabling pain |

Clinical abnormalities not mentioned in the above table can be graded according to the following adverse reaction criteria:

| **Grade 1** | **Grade 2** | **Grade 3** | **Grade 4** | **Grade 5** |
|---|---|---|---|---|
| Mild, short-term discomfort (<48 h), does not require medical treatment; | Mild-moderate to moderate, limits activities of daily life, does not require or only requires minimal medical intervention; | highly limits activities of daily life, requires daily care, requires medical treatment, and may require hospitalization; | Life-threatening, extremely limits activities of daily life, highly requires daily care, and requires medical treatment and hospitalization. | Death |

### 3.6 Causality Assessment of Adverse Event

The investigator should have measures in place to judge the causal relationship between solicited/unsolicited AEs and immunization, to identify immunization-related SAEs, and to suspend or terminate the clinical trial in a timely manner in order to minimize damages to the subjects.

General rules for causality assessment:

- ✓ **Definitely related:** Evidence of investigational vaccine immunization; AE follows a reasonable temporal sequence from immunization with the investigational vaccine; occurrence of AE is more reasonably explained by the investigational vaccine than other reasons; AE is observed after repeated immunization with the investigational vaccine; AE is consistent with what is known about this type of vaccine.
- ✓ **Probably related:** Evidence of investigational vaccine immunization; AE follows a reasonable temporal sequence from immunization with the investigational vaccine; occurrence of AE is more reasonably explained by the investigational vaccine than other reasons.
- ✓ **Possibly related:** Evidence of investigational vaccine immunization; AE follows a reasonable temporal sequence from immunization with the investigational vaccine; occurrence of AE may be explained by the investigational vaccine or other reasons.
- ✓ **Possibly unrelated:** Evidence of investigational vaccine immunization; AE is more likely caused by other reasons; AE is absent or undetermined after repeated immunization.
- ✓ **Definitely unrelated:** Absence of investigational vaccine immunization; or AE does not follow a reasonable sequence from immunization with the investigational vaccine; or AE is caused by other obvious reasons.

### 3.7 Sample Size Estimation

The sample size was estimated using an objective performance criteria method. Assuming the target value was 70% of the seroconversion rate of anti-pertussis in the co-purified DTaP group after vaccination, with a two-sided α of 0.05, a power of 90%, and a potential loss to follow-up of 15%, we estimated at least a sample size of 240 participants per group.

## 4. INCLUSION, EXCLUSION, TERMINATION, AND WITHDRAWAL CRITERIA

### 4.1 Inclusion Criteria

Subjects must meet the following criteria to be included in this study:

1) Age 6 years on the day of enrollment;
2) has signed and dated the informed consent form;

3) able to participate in all scheduled visits and comply with all study procedures (E.g. Completion of diary card and participation in visits);
4) received 4 doses of diphtheria, tetanus, and pertussis vaccine; not vaccinated with diphtheria, tetanus, and pertussis immunization products in the past 3 years;
5) had no history of pertussis, diphtheria, or tetanus;
6) ≥14 d since the last vaccine immunization;
7) body temperature ≤ 37.3°C confirmed by medical history and clinical examination before entering into this study.

**4.2 Exclusion Criteria**

**Exclusion criteria for first dose**

- allergy to any ingredient of the vaccine;
- past history of serious allergies to any immunization;
- medical or family history of convulsion, epilepsy, cerebral diseases and mental diseases;
- patient with immunodeficiency, has received immunosuppressants (oral steroids) during the treatment of malignant tumors or has HIV-induced immune impairment, or presence of congenital immune disease in close family member;
- injection of non-specific immunoglobulin within 1 month before enrollment;
- has acute febrile and infectious diseases;
- definite diagnosis of thrombocytopenia or history of other coagulopathy that may cause subcutaneous injection to be contraindicated;
- patient with serious chronic disease or in the acute phase of a chronic disease, or with uncontrolled hypertension or diabetes;
- various infectious, purulent, and allergic skin diseases;
- any other conditions that the investigator determines may impact the trial assessments.

**4.3 Termination and Removal Criteria**

Subject participation will be terminated early if any of the following is observed:

- protocol violations that render the investigator to believe that participation should be terminated;
- subject requests for trial withdrawal;
- intolerable AE, regardless of its relationship with the investigational vaccine;

- subject’s health conditions do not allow him/her to continue participating in the trial;
- any other reasons determined by the investigator.

### 4.4 Subject Withdrawal

Subject withdrawal refers to voluntary study withdrawal due to the subject’s inability to participate in the scheduled visits (AE/non-AE) or the inability to contact the subject during the visit scheduled by the study protocol.

Subjects will be informed that they have the right to withdraw from the study at any time and withdrawn subjects will not be replaced.

The investigator should try his/her best to reach the subjects who are unable to return for a visit at the scheduled time. All data should be collected from the subjects before the day of withdrawal for subsequent analysis. The date and possible reason for withdrawal should be documented in the source record and case report form (CRF):

- ■ SAE (name)
- ■ Non-SAE (name)
- ■ Protocol violation (detailed description)
- ■ Informed consent withdrawal not caused by AE
- ■ Departure from the location of the study site
- ■ Loss to follow-up
- ■ Death (collection of relevant information and certificate of the cause of death)
- ■ Other (detailed description)

## 5. PROTOCOL VIOLATIONS AND DEVIATIONS

### 5.1 Protocol Violations

- ✓ The investigator did not perform proper informed consent process with the subject;
- ✓ the investigator has violated the inclusion/exclusion criteria and enrolled ineligible subjects;
- ✓ the investigator has immunized the subject with a vaccine of the wrong arm or number;
- ✓ the investigator has immunized the subject with an improperly stored vaccine;
- ✓ other circumstances judged by the investigator that significantly impact the immunogenicity and safety assessments of the vaccine.

The investigator should immediately report protocol violations to the principal investigator and sponsor, pay close attention to the involved subjects, collect safety information, ensure the subjects' safety, and record the events in detail. Subjects with protocol violations can be withdrawn from the study upon the judgment of the principal investigator and sponsor.

**5.2 Protocol Deviations**

- ✓ Procedures are completed beyond the set time window (+ 14 d for blood collection);
- ✓ subject has poor adherence;
- ✓ subject has not completed blood sample collection;
- ✓ subject has taken prohibited medications (received ≥2 mg/kg/d intramuscular, oral or intravenous systemic corticosteroids for ≥14 d or other immunosuppressants);
- ✓ insufficient time since immunization with other vaccines.

The investigator should record all protocol deviations in detail and report them to the principal investigator and sponsor. The principal investigator and sponsor will then determine whether the subject can proceed with the rest of the study. In addition, the principal investigator should assess the subject's data to determine whether they can be included in the safety or immunogenicity analysis.

## 6. ANALYSIS SETS

**Full analysis set (FAS):**

The FAS is the ideal subject population determined based on the intention to treat (ITT) principle, which includes all randomized subjects who have received at least one dose of the vaccine and have valid pre-immunization antibody titer results.

**Per-protocol set (PPS):**

The PPS is a subset of the FAS that contains subjects with better protocol adherence. All subjects who conform to the inclusion/exclusion criteria, have completed all visits as required by the protocol, and were not removed after blinded review of the serology results are included in the PPS.

Subjects who meet the following conditions are not included in the PPS:

- ➢ Subjects without valid pre- and post-immunization antibody titer values;
- ➢ subjects with incomplete immunization;

- subjects with blood samples collected beyond the set time window;
- subjects immunized with the wrong vaccine;
- received immunoglobulin injection or whole blood/plasma infusion after immunization to post-immunization blood collection.

PPS is the primary analysis set in this study. Explanation is required if inconsistency is identified between the FAS and PPS.

**Safety set (SS):**

All randomized subjects who have been immunized with one dose of the investigational vaccine and have at least one safety assessment are included in the SS. The SS is primarily used for the analysis of vaccine-related AEs, SAEs, and concomitant medications.

# CONSORT 2010 checklist of information to include when reporting a randomised trial*

| Section/Topic | Item No | Checklist item | Reported on page No |
|---|---|---|---|
| **Title and abstract** | | | |
| | 1a | Identification as a randomised trial in the title | 1 |
| | 1b | Structured summary of trial design, methods, results, and conclusions (for specific guidance see CONSORT for abstracts) | 2 |
| **Introduction** | | | |
| Background and objectives | 2a | Scientific background and explanation of rationale | 3-4 |
| | 2b | Specific objectives or hypotheses | 4 |
| **Methods** | | | |
| Trial design | 3a | Description of trial design (such as parallel, factorial) including allocation ratio | 12 |
| | 3b | Important changes to methods after trial commencement (such as eligibility criteria), with reasons | NA |
| Participants | 4a | Eligibility criteria for participants | 12 |
| | 4b | Settings and locations where the data were collected | 12 |
| Interventions | 5 | The interventions for each group with sufficient details to allow replication, including how and when they were actually administered | 14-15 |
| Outcomes | 6a | Completely defined pre-specified primary and secondary outcome measures, including how and when they were assessed | 15 |
| | 6b | Any changes to trial outcomes after the trial commenced, with reasons | NA |
| Sample size | 7a | How sample size was determined | 15 |
| | 7b | When applicable, explanation of any interim analyses and stopping guidelines | NA |
| Randomisation: | | | |
| Sequence generation | 8a | Method used to generate the random allocation sequence | 13 |
| | 8b | Type of randomisation; details of any restriction (such as blocking and block size) | 13 |
| Allocation concealment mechanism | 9 | Mechanism used to implement the random allocation sequence (such as sequentially numbered containers), describing any steps taken to conceal the sequence until interventions were assigned | 13 |
| Implementation | 10 | Who generated the random allocation sequence, who enrolled participants, and who assigned participants to interventions | 13 |
| Blinding | 11a | If done, who was blinded after assignment to interventions (for example, participants, care providers, those | NA |

| | | | |
|---|---|---|---|
| | | assessing outcomes) and how | |
| | 11b | If relevant, description of the similarity of interventions | NA |
| Statistical methods | 12a | Statistical methods used to compare groups for primary and secondary outcomes | 15-16 |
| | 12b | Methods for additional analyses, such as subgroup analyses and adjusted analyses | NA |
| **Results** | | | |
| Participant flow (a diagram is strongly recommended) | 13a | For each group, the numbers of participants who were randomly assigned, received intended treatment, and were analysed for the primary outcome | 5, Figure 1 |
| | 13b | For each group, losses and exclusions after randomisation, together with reasons | 5, Figure 1 |
| Recruitment | 14a | Dates defining the periods of recruitment and follow-up | 5 |
| | 14b | Why the trial ended or was stopped | NA |
| Baseline data | 15 | A table showing baseline demographic and clinical characteristics for each group | 5, Table1 |
| Numbers analysed | 16 | For each group, number of participants (denominator) included in each analysis and whether the analysis was by original assigned groups | 5 |
| Outcomes and estimation | 17a | For each primary and secondary outcome, results for each group, and the estimated effect size and its precision (such as 95% confidence interval) | 5-6 |
| | 17b | For binary outcomes, presentation of both absolute and relative effect sizes is recommended | 5-6 |
| Ancillary analyses | 18 | Results of any other analyses performed, including subgroup analyses and adjusted analyses, distinguishing pre-specified from exploratory | NA |
| Harms | 19 | All important harms or unintended effects in each group (for specific guidance see CONSORT for harms) | 6 |
| **Discussion** | | | |
| Limitations | 20 | Trial limitations, addressing sources of potential bias, imprecision, and, if relevant, multiplicity of analyses | 11 |
| Generalisability | 21 | Generalisability (external validity, applicability) of the trial findings | 8-10 |
| Interpretation | 22 | Interpretation consistent with results, balancing benefits and harms, and considering other relevant evidence | 11-12 |
| **Other information** | | | |
| Registration | 23 | Registration number and name of trial registry | 12 |
| Protocol | 24 | Where the full trial protocol can be accessed, if available | 16 |
| Funding | 25 | Sources of funding and other support (such as supply of drugs), role of funders | 20 |

*We strongly recommend reading this statement in conjunction with the CONSORT 2010 Explanation and Elaboration for important clarifications on all the items. If relevant, we also recommend reading CONSORT extensions for cluster randomised trials, non-inferiority and equivalence trials, non-pharmacological treatments, herbal interventions, and pragmatic trials. Additional extensions are forthcoming: for those and for up to date references relevant to this checklist, see www.consort-statement.org.
