# Supplementary material for: Immunogenicity and safety of co-purified diphtheria, tetanus and acellular pertussis vaccine in 6-year-old Chinese children
Source: Nat Commun. 2025 Nov 29;17:214. doi: 10.1038/s41467-025-66890-7 (PMC12780235; doi:10.1038/s41467-025-66890-7)
Supplement: Supplementary file 3 — Reporting summary [file 41467_2025_66890_MOESM3_ESM.pdf]

nature portfolio

Corresponding author(s): Hanqing He

Last updated by author(s): Nov 6, 2025

Reporting Summary

Nature Portfolio wishes to improve the reproducibility of the work that we publish. This form provides structure for consistency and transparency in reporting. For further information on Nature Portfolio policies, see our Editorial Policies and the Editorial Policy Checklist.

Statistics

For all statistical analyses, confirm that the following items are present in the figure legend, table legend, main text, or Methods section.

n/a	Confirmed	
☐	☒	The exact sample size (*n*) for each experimental group/condition, given as a discrete number and unit of measurement
☐	☒	A statement on whether measurements were taken from distinct samples or whether the same sample was measured repeatedly
☐	☒	The statistical test(s) used AND whether they are one- or two-sided<br>*Only common tests should be described solely by name; describe more complex techniques in the Methods section.*
☒	☐	A description of all covariates tested
☒	☐	A description of any assumptions or corrections, such as tests of normality and adjustment for multiple comparisons
☐	☒	A full description of the statistical parameters including central tendency (e.g. means) or other basic estimates (e.g. regression coefficient) AND variation (e.g. standard deviation) or associated estimates of uncertainty (e.g. confidence intervals)
☐	☒	For null hypothesis testing, the test statistic (e.g. *F*, *t*, *r*) with confidence intervals, effect sizes, degrees of freedom and *P* value noted<br>*Give P values as exact values whenever suitable.*
☒	☐	For Bayesian analysis, information on the choice of priors and Markov chain Monte Carlo settings
☒	☐	For hierarchical and complex designs, identification of the appropriate level for tests and full reporting of outcomes
☒	☐	Estimates of effect sizes (e.g. Cohen's *d*, Pearson's *r*), indicating how they were calculated

Our web collection on statistics for biologists contains articles on many of the points above.

Software and code

Policy information about availability of computer code

Data collection	The collected data were entered into Epi-data version 3.1.
Data analysis	The statistical analysis was performed using SAS version 9.4.

For manuscripts utilizing custom algorithms or software that are central to the research but not yet described in published literature, software must be made available to editors and reviewers. We strongly encourage code deposition in a community repository (e.g. GitHub). See the Nature Portfolio guidelines for submitting code & software for further information.

Data

Policy information about availability of data

All manuscripts must include a data availability statement. This statement should provide the following information, where applicable:

- Accession codes, unique identifiers, or web links for publicly available datasets
- A description of any restrictions on data availability
- For clinical datasets or third party data, please ensure that the statement adheres to our policy

The study protocol is available in the Supplementary Information file. Individual participant data are available under restricted access for the requirements imposed by the Ethics Committee of Zhejiang Provincial Center for Disease Control and Prevention. Researchers who provide a scientifically sound proposal Data and sign a data access agreement will be allowed access to the de-identified individual participant data. Individual participant data can be shared through contacting the corresponding authors.

Research involving human participants, their data, or biological material

Policy information about studies with human participants or human data. See also policy information about sex, gender (identity/presentation), and sexual orientation and race, ethnicity and racism.

Reporting on sex and gender	This study included both female and male children aged 6 to 7 years in Zhejiang Province, China. The parent or legal guardian of each participant self-reported the sex. There were 53.33% female participants in the DTaP and 52.08% female participants in the DT (P>0.05). No sensitivity analyses stratified by sex were performed.
Reporting on race, ethnicity, or other socially relevant groupings	All participants were of Asian race, with 477 (>99%) being of Han Chinese ethnicity.
Population characteristics	The mean age was 73.49 months in the DTaP and 73.43 months in the DT. There were 128 (53.33%) female participants in the DTaP and 125 (52.08%) female participants in the DT. All participants were of Asian race, with 477 (>99%) being of Han Chinese ethnicity. Demographic and baseline characteristics of the participants were generally well balanced between the two arm.
Recruitment	Between April 2023 and May 2024, 527 participants were assessed for eligibility and 480 participants randomly assigned (1:1) to either the DTaP or DT arms. The participants were enrolled from two sites (Fuyang District of Hangzhou City and Tongxiang City of Zhejiang Province, China).although guardians were asked to self-report a history of pertussis at enrollment, active surveillance for pertussis-like symptoms was not conducted in participants or their close contacts prior to enrollment, and participants were not screened for pertussis infection based on a standardized case definition (such as laboratory confirmation, clinical criteria, and/or epidemiological links) before vaccination. As a result, some participants with asymptomatic or moderate symptomatic pertussis infection who did not visit health care facilities may have been recruited in the trial, which could have confounded the immunogenicity results.
Ethics oversight	This trial complies with all relevant ethical regulations, and the protocol, informed consent, and amendments was approved by the Ethics Committee of Zhejiang Provincial Center for Disease Control and Prevention (2023-008-1). This trial is conducted in accordance with the Declaration of Helsinki and Good Clinical Practice guidelines. Informed consent, signed by a parent or legal guardian, was obtained from all participants before any study procedure.

Note that full information on the approval of the study protocol must also be provided in the manuscript.

Field-specific reporting

Please select the one below that is the best fit for your research. If you are not sure, read the appropriate sections before making your selection.

☒ Life sciences ☐ Behavioural & social sciences ☐ Ecological, evolutionary & environmental sciences

For a reference copy of the document with all sections, see nature.com/documents/nr-reporting-summary-flat.pdf

Life sciences study design

All studies must disclose on these points even when the disclosure is negative.

Sample size	The sample size was estimated using an objective performance criteria method. Assuming the target value was 70% of the seroconversion rate of anti-pertussis in the DTaP group after vaccination, with a two-sided α of 0.05, a power of 90%, and a potential loss to follow-up of 15%, we estimated at least a sample size of 240 participants per group.
Data exclusions	Immunogenicity was assessed in the per-protocol set (PPS), which included all participants who met the inclusion/exclusion criteria and completed vaccination, follow-up, and blood collections as required by the protocol. The safety set (SS) included all participants who had received at least one dose of vaccination.
Replication	No replication had been performed in this study due to the study design (a randomised, controlled trial).We disclosed the characteristics of the study population, the study location, the trial methods, the vaccine manufacturer and batch number, the immunogenicity testing methods and the testing party, as well as the content of data statistical analysis, for the reproducibility of the research.
Randomization	Participants were randomly (1:1) assigned to receive either DTaP (DTaP arm) or DT (DT arm). Randomization was performed using a block randomization method with a block size of 8, and random number tables were generated by a statistician using SAS 9.4.
Blinding	The study was an open-label trial. Participants and investigators were not blinded to the group assignment, but laboratory technicians were blinded to the vaccines received in each group.

Reporting for specific materials, systems and methods

We require information from authors about some types of materials, experimental systems and methods used in many studies. Here, indicate whether each material, system or method listed is relevant to your study. If you are not sure if a list item applies to your research, read the appropriate section before selecting a response.

Materials & experimental systems

n/a	Involved in the study	
☒	☐	Antibodies
☒	☐	Eukaryotic cell lines
☒	☐	Palaeontology and archaeology
☒	☐	Animals and other organisms
☐	☒	Clinical data
☒	☐	Dual use research of concern
☒	☐	Plants

Methods

n/a	Involved in the study	
☒	☐	ChIP-seq
☒	☐	Flow cytometry
☒	☐	MRI-based neuroimaging

Clinical data

Policy information about clinical studies

All manuscripts should comply with the ICMJE guidelines for publication of clinical research and a completed CONSORT checklist must be included with all submissions.

Clinical trial registration	This trial was registered with ClinicalTrials.gov (NCT05870631).
Study protocol	The protocol has been submitted to the Nature Communications on-line submission system.
Data collection	The data was collected at the Fuyang District of Hangzhou City and Tongxiang City of Zhejiang Province, China. Participants were recruited from April to July 2023. Data collection was completed by May 2024.
Outcomes	The primary outcomes of the study were the seroconversion rates and seropositive rates of anti-pertussis antibodies in the DTaP group, as well as a comparison of seroconversion rates and seropositive rates of anti-diphtheria and anti-tetanus antibodies in the DTaP and DT groups. The secondary outcome was the rates of adverse events 28 days after immunization with the DTaP or DT, as well as antibody geometric mean concentrations (GMCs) against pertussis, diphtheria, and tetanus before and after vaccination.

Plants

Seed stocks	*Report on the source of all seed stocks or other plant material used. If applicable, state the seed stock centre and catalogue number. If plant specimens were collected from the field, describe the collection location, date and sampling procedures.*
Novel plant genotypes	*Describe the methods by which all novel plant genotypes were produced. This includes those generated by transgenic approaches, gene editing, chemical/radiation-based mutagenesis and hybridization. For transgenic lines, describe the transformation method, the number of independent lines analyzed and the generation upon which experiments were performed. For gene-edited lines, describe the editor used, the endogenous sequence targeted for editing, the targeting guide RNA sequence (if applicable) and how the editor was applied.*
Authentication	*Describe any authentication procedures for each seed stock used or novel genotype generated. Describe any experiments used to assess the effect of a mutation and, where applicable, how potential secondary effects (e.g. second site T-DNA insertions, mosiacism, off-target gene editing) were examined.*
